# Draft Genome Sequences of Vibrio vulnificus Strains Recovered from Moribund Tilapia

**DOI:** 10.1128/MRA.00094-21

**Published:** 2021-06-03

**Authors:** Héctor Carmona-Salido, Belén Fouz, Eva Sanjuán, Miguel Carda-Diéguez, Christian M. J. Delannoy, Neris García-González, Fernando González-Candelas, Carmen Amaro

**Affiliations:** a Departamento de Microbiología y Ecología, Universitat de València, Burjassot, Valencia, Spain; b Instituto Universitario de Biotecnología y Biomedicina BIOTECMED, Universitat de València, Burjassot, Valencia, Spain; c Skretting Group, Stavanger, Norway; d Institute for Integrative Systems Biology, I2SysBio (CSIC-UV), University of Valencia, Valencia, Spain; e Infection and Public Health Joint Research Unit, FISABIO-University of Valencia, Valencia, Spain; f CIBER in Epidemiology and Public Health, Valencia, Spain; University of Delaware

## Abstract

Potentially zoonotic Vibrio vulnificus strains were isolated from vibriosis outbreaks occurring on eastern Mediterranean tilapia farms between 2016 and 2019. In this work, the draft genome sequences of three representative isolates are presented.

## ANNOUNCEMENT

Vibrio vulnificus is a zoonotic pathogen that inhabits marine and estuarine ecosystems in tropical, subtropical, and temperate zones (1, 2). This pathogen is currently spreading to colder areas due to climate change (1, 3–5). In both fish and humans, this bacterium causes a range of diseases known worldwide as vibriosis (1, 6, 7). The severity of these manifestations is increased in fishes raised in brackish water above 25°C (7, 8).

Major vibriosis outbreaks are frequent over the summer months in tilapia pond culture in eastern Mediterranean countries. Several outbreaks were registered on two tilapia farms from 2016 to 2019. The tilapia showed clinical signs of a hemorrhagic septicemia. Samples from the eyes, brain, and kidney of moribund tilapia (400 g) (Table 1) were directly streaked onto a blood agar base (Oxoid Ltd., Basingstoke, UK) with 5% citrated calf blood. Pure cultures were obtained after incubation at 28°C for 48 h. The isolated bacteria were identified as V. vulnificus using an API-20E system (bioMérieux, Spain) and using PCR with primers for *vvha* (9–11). For sequencing purposes, we selected strains Vv5, Vv3, and TI417.

**TABLE 1:** NCBI accession numbers and *de novo* assembly statistics for clade T strains obtained using QUAST software

Strain name	No. of contigs	Total length (bp)	Largest contig (bp)	GC (%)	*N*_50_ (bp)	*N*_75_ (bp)	*L*_50_	*L*_75_	Genome coverage (×)	No. of genes	BioSample accession no.	GenBank accession no.	SRA accession no.	Tissue source
Vv5	16	5,305,952	2,909,025	46.64	2,909,025	1,771,586	1	2	88.7	4,648	SAMN16587835	JADIXT000000000	SRR13236868, SRX9866694	Kidney
Vv3	216	5,204,182	383,328	46.61	108,105	65,177	15	31	57.7	4,833	SAMN16587836	JADIXU000000000	SRR13236867	Eyes
TI417	587	5,399,057	352,890	46.38	98,248	58,826	17	34	88.01	5,058	SAMN16587837	JADIXV000000000	SRR13236866	Brain

The selected strains were grown in Luria-Bertani broth (LB-1) overnight. After that, DNA was extracted using a GenElute bacterial genomic DNA kit (Sigma) following the manufacturer’s instructions. The DNA integrity was checked using electrophoresis and NanoDrop technology. The DNA was quantified using an Invitrogen Qubit 3.0 fluorometer (Thermo Fisher Scientific, USA). Next, the DNA was sequenced using Illumina MiSeq technology. The Illumina library construction and sequencing of Vv5 were performed by SCSIE (Servei Central de Suport a la Investigació Experimental), University of Valencia, using an Illumina TruSeq DNA PCR-free sample prep kit following the manufacturer’s instructions, which yielded 250-bp paired-ends reads. Next, we attempted to close the genome sequence of strain Vv5. To this end, the genome was sequenced using an Oxford Nanopore MinION device. The MinION library construction and sequencing were performed by FISABIO-University of Valencia's sequencing service, using the Oxford Nanopore PCR barcoding kit (SQK-PBK004), following the manufacturer’s instructions.

On the other hand, the library construction and sequencing of Vv3 and TI417 were performed by FISABIO’s sequencing service, using the Illumina (San Diego, CA, USA) NextSeq platform with a Nextera XT library preparation kit and following the manufacturer’s protocols, which generated 150-bp paired-end reads. For strains Vv3 and TI417, no Oxford Nanopore sequencing was performed.

The short reads were quality filtered using PRINSEQ (12). For the Vv5 hybrid assembly, we performed *de novo* assembly using Unicycler v. 0.4.9b (13) with default parameters and normal mode. The short reads were *de novo* assembled using SPAdes v. 3.13 (14) with careful mode and default options. The draft genome sequences were annotated using the NCBI Prokaryotic Genome Annotation Pipeline (PGAP) v. 4.13 (15). The assembly statistics were retrieved using QUAST v. 5 (16) (default, adding --gene-finding option) and PGAP. The genome completeness was assessed using BUSCO (17). We selected genome mode and the auto-lineage option, obtaining values of 99.8% complete benchmarking universal single-copy orthologs (BUSCOs) for all isolates. Table 1 shows the assembly statistics for each strain genome. Finally, we calculated the average nucleotide identity (ANI) using strain Vv5 as a reference. The values were close to 100% (99.95% for TI417 and 99.97% for Vv3) compared with strains of the same group, whereas the highest value compared with other strains of this species was 98.20% with yb158 strain (18).

### Data availability.

This whole-genome shotgun project has been deposited at DDBJ/ENA/GenBank under the accession numbers JADIXT000000000, JADIXU000000000, and JADIXV000000000. The versions described in this paper for Vv3 and TI417 are the first versions JADIXU010000000 and JADIXV010000000, respectively, and for Vv5, the second version, JADIXT020000000. All samples are collected under the BioProject accession number PRJNA673082. The SRA accession numbers for the paired-end reads are SRR13236868, SRR13236867, and SRR13236866. The SRA accession number for the MinION reads is SRX9866694.
